# Evolution of complexity in non-viral oligonucleotide delivery systems: from gymnotic delivery through bioconjugates to biomimetic nanoparticles

**DOI:** 10.1080/15476286.2022.2147278

**Published:** 2022-11-21

**Authors:** Kamil Bakowski, Stefan Vogel

**Affiliations:** Department of Physics, Chemistry and Pharmacy, University of Southern Denmark, Odense, Denmark

**Keywords:** RNA, delivery, oligonucleotides, nucleic acid chemistry, drug development

## Abstract

From the early days of research on RNA biology and biochemistry, there was an interest to utilize this knowledge and RNA itself for therapeutic applications. Today, we have a series of oligonucleotide therapeutics on the market and many more in clinical trials. These drugs - exploit different chemistries of oligonucleotides, such as modified DNAs and RNAs, peptide nucleic acids (PNAs) or phosphorodiamidate morpholino oligomers (PMOs), and different mechanisms of action, such as RNA interference (RNAi), targeted RNA degradation, splicing modulation, gene expression and modification. Despite major successes e.g. mRNA vaccines developed against SARS-CoV-2 to control COVID-19 pandemic, development of therapies for other diseases is still limited by inefficient delivery of oligonucleotides to specific tissues and organs and often prohibitive costs for the final drug. This is even more critical when targeting multifactorial disorders and patient-specific biological variations. In this review, we will present the evolution of complexity of oligonucleotide delivery methods with focus on increasing complexity of formulations from gymnotic delivery to bioconjugates and to lipid nanoparticles in respect to developments that will enable application of therapeutic oligonucleotides as drugs in personalized therapies.

## Brief history of the RNA therapeutics

**Figure 1.**
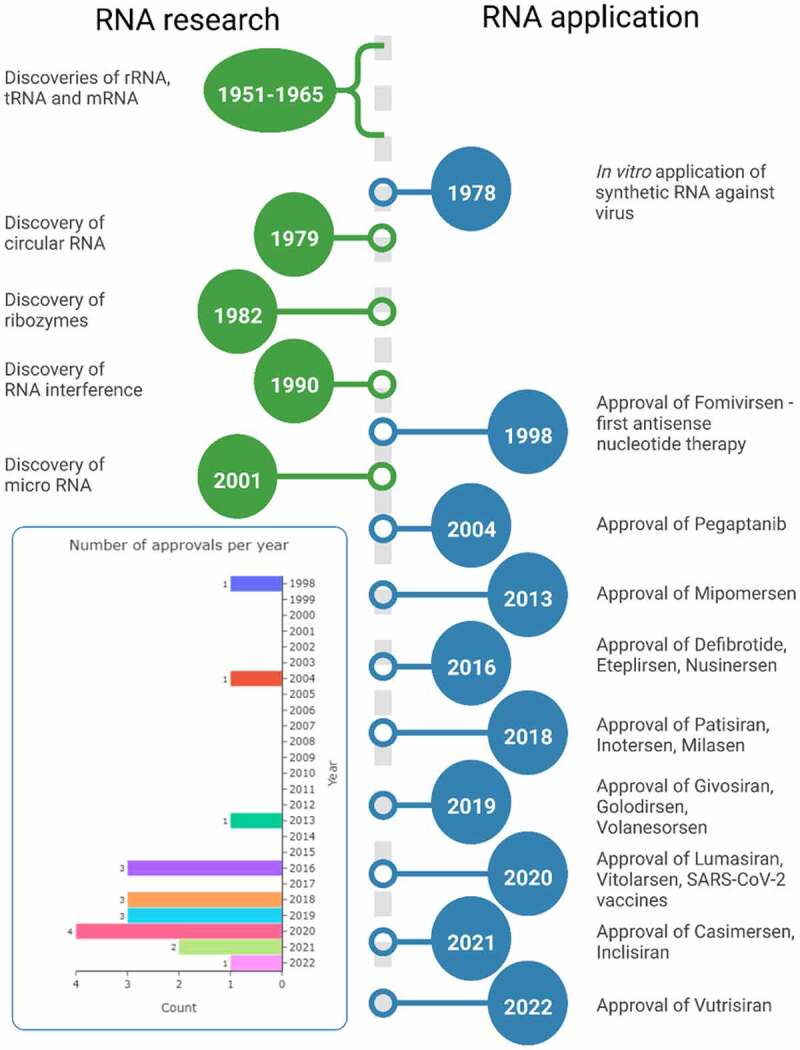
Timeline of RNA research and its therapeutic applications (not to time scale). In the insert showed number of approved oligonucleotide drugs per year (1998–2022).

**Figure 2.**
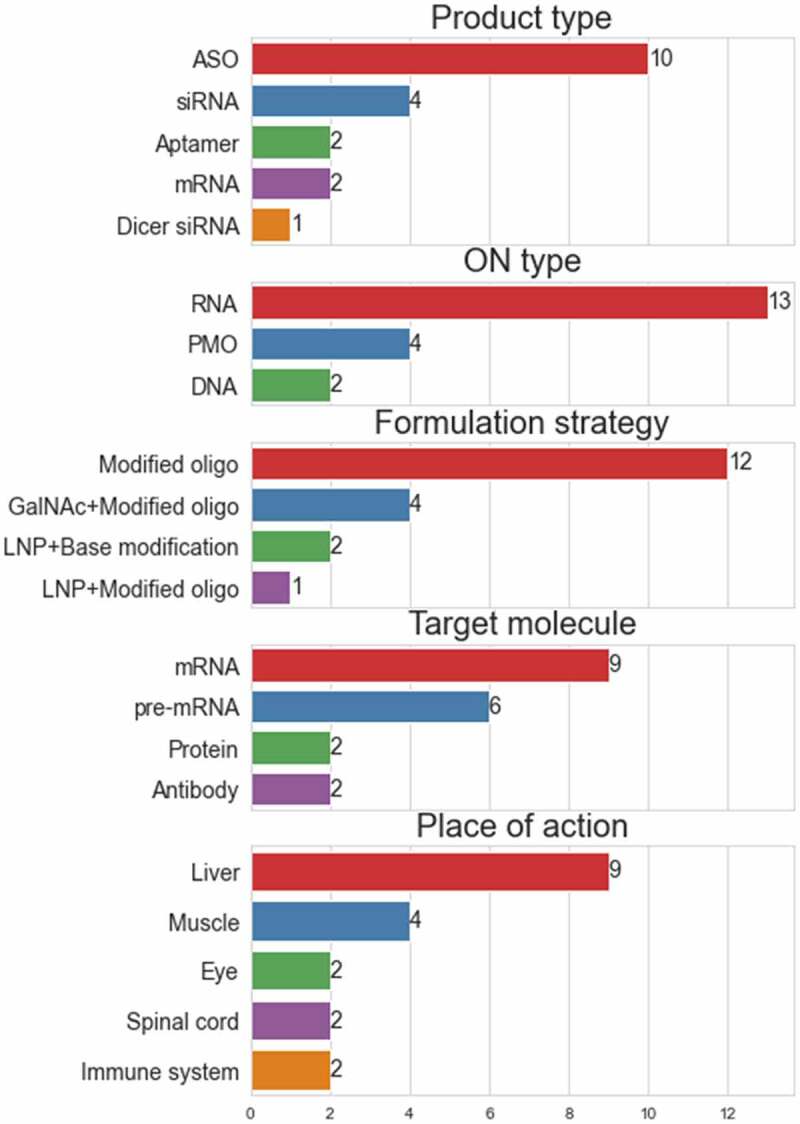
Summary of features of FDA approved drugs. Bars and numbers show total counts for each feature as shown in Table 1. Each bar graph represents one of the columns.

The development of oligonucleotide therapeutics is tightly connected to the history of RNA research, and both have complicated stories. In the late 19th and early 20th centuries, researchers defined the basic differences between DNA and RNA, but a functional characterization of RNA is still an ongoing process, with new functions of RNAs in cellular processes still being discovered (Figure 1 – discoveries vs approval line-up)[1]. Although many discoveries can be traced to a number seminal papers, the discovery of mRNA was a long effort of many research groups decoding this complex process. This is relevant when considering therapeutic applications, as almost all oligonucleotide therapies, currently approved or in clinical trials, target mRNA, pre-mRNA or use mRNA (Figure 2 and Table 1 – FDA approved drugs) [1]. The complexity of RNA regulation results in major design challenges for new therapeutic oligonucleotides. Early-stage oligonucleotide (ON) therapies, e.g. Fomivirsen, Pegatanib, were very expensive, and although at the time of approval offering unique treatment, they were later outcompeted by small-molecule therapies[2]. In the early 2000s, the high production costs and discouraging results from clinical trials caused withdrawal of investors, which held back further clinical translation[3]. At that time, effective strategies for sequence selection and techniques of oligonucleotide delivery were not developed enough, which both contributed to low efficiency and serious side effects from off-target effects observed during clinical trials. Nowadays, advanced digital tools help with the design of ONs with high binding efficiency and low off-target effects[4]. In parallel, development and adoption of new building blocks such as substituted riboses e.g. 2’-O-methyl (2’-Ome), 2’-O-methoxyethyl (2’-MOE), ribose cyclization between 2’ and 5’ position e.g. locked nucleic acids (LNAs) and alternative oligonucleotide backbone chemistry e.g. phosphorothioates, phosphorodiamidate morpholino oligomers (PMOs) and peptide nucleic acids (PNAs), greatly improved oligonucleotides serum stability and binding specificity. These developments led to new drug approvals and regained interest from investors, with steady approval rate of 2–3 drugs per year, which is impressive by current standards in pharmaceutical industry (Figure 1 – approvals per year). The review is focused on the evolution of non-viral oligonucleotide delivery systems and proposes a classification that includes past, current and potential future developments. The classification is based on the increasing level of molecular complexity in response to increased demands in respect to functionality, with functionality describing both the mode of action on the biological target and required auxiliary functionality such as specific receptor targeting ligands, cell-penetrating moieties, Albumin binding and other functionalities required for efficient delivery and bioactivity of a given therapeutic oligonucleotide. At the time of writing, there are over 850 ON therapies at preclinical and clinical stage, which is beyond the scope of this review. The cited research is at primarily at preclinical stage with a focus on available chemistry platforms and different formulation strategies including gymnotic delivery, bioconjugates and nanoparticle-based formulations. The numerical analysis was limited to approved drugs to show progress achieved with currently available formulation strategies.

**Table 1.** Simplified categorization of properties of FDA approved oligonucleotide therapies.

Product name	Year of Approval	Product type	ON type	Route of delivery	Formulation strategy	Target molecule	Site of action	Indication	Company
Fomivirsen	1998	ASO	DNA	IVT	Modified oligo	mRNA	Eye	Cytomegalovirus retinitis	Ionis Pharma/Novartis
Pegaptanib	2004	Aptamer	RNA	IVT	Modified oligo	Protein	Eye	Neovascular age-related macular degeneration	NeXstar Pharma/Eyetech Pharma
Mipomersen	2013	ASO	RNA	SQ	Modified oligo	mRNA	Liver	Homozygous familial hypercholesterolemia	Ionis Pharma/Genzyme/Kastle Tx
Defibrotide	2016	Aptamer	DNA	IV	Modified oligo	Protein	Liver	Hepatic veno-occlusive disease	Jazz Pharma
Eteplirsen	2016	ASO	PMO	IV	Modified oligo	pre-mRNA	Muscle	Duchenne muscular dystrophy	Sarepta Therapeutics
Nusinersen	2016	ASO	RNA	IT	Modified oligo	pre-mRNA	Spinal cord	Spinal muscular atrophy	Ionis Pharma/Biogen
Patisiran	2018	siRNA	RNA	IV	LNP+Modified oligo	mRNA	Liver	Hereditary transthyretin amyloidosis,polyneuropathy	Alnylam Pharma
Inotersen	2018	ASO	RNA	SQ	Modified oligo	mRNA	Liver	Hereditary transthyretin amyloidosis,polyneuropathy	Ionis Pharma/Akcea Pharam
Milasen	2018	ASO	RNA	IT	Modified oligo	pre-mRNA	Spinal cord	Mila Makovec’s CLN7 gene associated with Batten disease	Boston Children’s Hospital
Volanesorsen	2019	ASO	RNA	SQ	Modified oligo	mRNA	Liver	Familial partial lipodystrophy	Ionis Pharma
Givosiran	2019	Dicer siRNA	RNA	SQ	GalNAc+Modified oligo	mRNA	Liver	Acute hepatic porphyria	Alnylam Pharma
Golodirsen	2019	ASO	PMO	IV	Modified oligo	pre-mRNA	Muscle	Duchenne muscular dystrophy	Sarepta Therapeutics
Vitolarsen	2020	ASO	PMO	IV	Modified oligo	pre-mRNA	Muscle	Duchenne muscular dystrophy	NS Pharma
Lumasiran	2020	siRNA	RNA	SQ	GalNAc+Modified oligo	mRNA	Liver	Primary hyperoxaluria type 1	Alnylam Pharma
BNT162b2	2020	mRNA	RNA	IM	LNP+Base modification	Antibody	Immune system	COVID-19	BioNTech/Pfizer
mRNA-1273	2020	mRNA	RNA	IM	LNP+Base modification	Antibody	Immune system	COVID-19	Moderna
Casimersen	2021	ASO	PMO	IV	Modified oligo	pre-mRNA	Muscle	Duchenne muscular dystrophy	Sarepta Therapeutics
Inclisiran	2021	siRNA	RNA	SQ	GalNAc+Modified oligo	mRNA	Liver	Atherosclerotic cardiovascular disease	Alnylam Pharma/Novartis
Vutrisiran	2022	siRNA	RNA	SQ	GalNAc+Modified oligo	mRNA	Liver	Hereditary transthyretin amyloidosis,polyneuropathy	Alnylam Pharma

a IVT – Intravitreal Injection, SQ – Subcutaneous, IV–Intravenous, IT – Intrathecal, IM – Intramuscular.

## Therapeutic targets and mechanisms of action

By analysing properties of ON drugs, currently approved and in clinical trials, general trends, strengths and limitations of applied strategies can be observed (Figure 2 and Table 1) [5,6]. Starting from the route of administration of ON drugs, there are two major categories, local administration through intravitreal or intrathecal injections and systemic delivery through intravenous or subcutaneous injections. The local administration routes help to avoid common ON degradation pathways and thereby improve ON stability and cell specificity by proximity to the target tissue. Intrathecal injections, although not necessarily locally targeted, are limiting potential target tissues and the exposure to the immune system. As for now, intrathecal injections are the only routes of administration targeting brain and CNS, although new strategies based on conjugated peptides, multifunctional lipid nanoparticles (LNP) [7] or extracellular vesicles (EVs) are being developed [8]. With systemic delivery, formulations target mostly liver cells, as Kupffer and liver sinusoidal endothelial cells by promoting nanoparticle extravasation and further clearance (Figure 2 – ‘Organ target’)

Although clearance pathways are well described and modelled for small molecules, this knowledge is only partially translatable to pharmacokinetic and pharmacodynamic modelling for ON-based therapeutics[9]. Some strategies for e.g. to avoid renal clearance also apply for ON formulations, by nanoparticle size restriction or PEGylation. Administration close to the site of action may also circumvent common clearance routes, as recently shown for mRNA vaccines developed against SARS-CoV-2 where local translation of corona proteins in muscular tissues influence the immune response of the whole body. Intracellularly, the majority of ON therapies are targeting mRNA and pre-mRNA, modulating final protein expression (Figure 2 – ‘Target’). To achieve that effect, antisense oligonucleotides (ASO) or small interfering RNAs (siRNA) are used. Chemical modifications of RNA at 2’ position and/or modifications of the phosphate backbone or conjugation to other building blocks (e.g. hydrophobic moieties or targeting carbohydrates) are essential for successful delivery and clinically relevant pharmacodynamics.

Frequently used phosphorothioate modifications improve cell penetration, lower nuclease mediated degradation and modulate hybridization to the target sequence[10]. Substitution of only a few nucleotides in siRNA by locked nucleic acids (LNAs) dramatically change the binding affinity[11], improve sequence specificity and *in vivo* stability [12]. Moreover, chemical modification of the ON backbone to PMO or PNA increases resistance towards most nuclease-based enzymatic degradation while maintaining a high level of biological functionality and activity [13,14]. Nucleic Acid based therapies rely on different mechanisms to engage with different molecular targets (Table 2) which each have intrinsic advantages and disadvantages, the same therapeutic target can often be adressed by more than one of the strategies shown in Table 2. However, independent of the mechanism of action and the strategy used, delivery of oligonucleotides remains a major challenge for the sucessful application of therapeutic oligonucleotides. The delivery challenges are primarily linked to the size of the oligonucleotide (length), the sequence design and most importantly to the chemistry used depending on the number and nature of modified non-natural building blocks incoporated (particular modifications are discussed more detailed in connection with different delivery strategies).

**Table 2.** Mechanisms of action and basic features of nucleic acid therapies.

ON	Size [nt]	Mechanism of action	Molecular target	Localization
ASO	13–25	Steric blocking of translation; Induction of RNase H-mediated degradation	Single or multiple mRNAs or pre-mRNAs	Cytosol, nucleus
siRNA	19–25	Induction of RISC-mediated degradation; Steric blocking of translation	Single mRNA	Cytosol
Dicer substrate siRNA	25–30	After processing by Dicer nuclease induction of RISC-mediated degradation or steric blocking of translation	Single mRNA	Cytosol
miRNA	20–24	Induction of RISC-mediated degradation; Steric blocking of translation	Multiple mRNAs	Cytosol
AntimiR and AntagomiR	20–25	Steric blocking of interaction	Single miRNA	Cytosol
Aptamer	50–120	Modification of protein activity by site-specific binding	Single or multiple peptides, proteins, polysaccharides	Cytosol, nucleus
mRNA	>200	Translation into peptides or proteins	Disruption or reconstitution of metabolic function; potentially very diverse interactions	Cytosol or any membrane

## Challenges in developing successful RNA therapy

**Figure 3.**
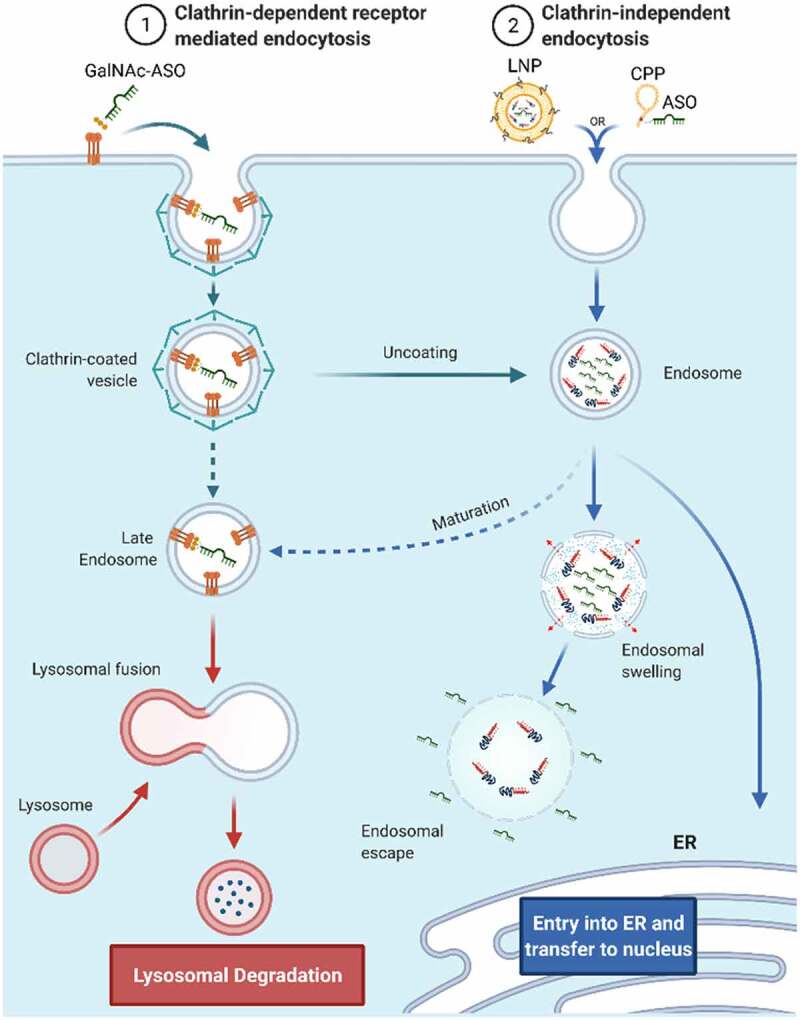
Schematic representation of therapeutic RNAs with cellular entry and trafficking.

**Figure 4.**
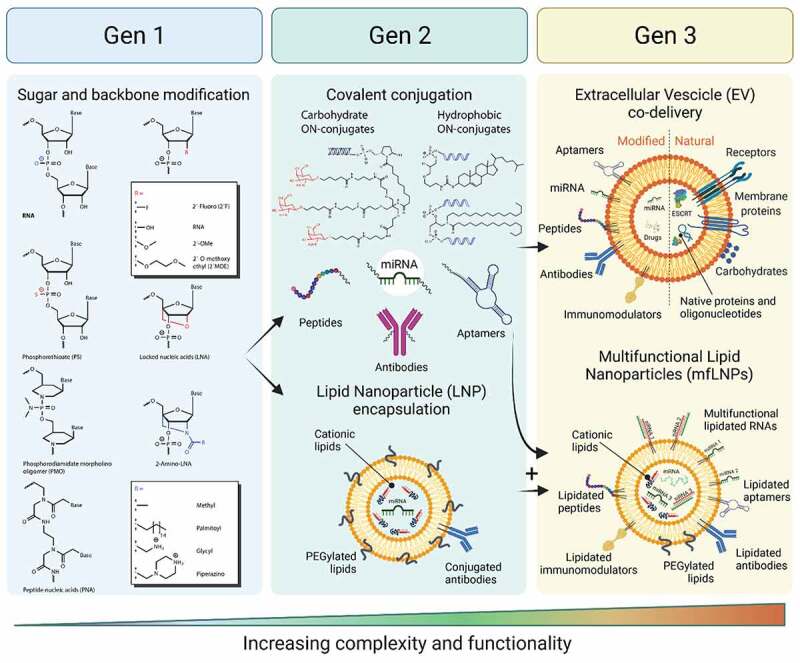
Categorization of RNA delivery systems into 3 generations.

The optimal delivery system for RNA-based therapies should allow for a simple, non-invasive route of delivery to the body, avoid unwanted immune response, allow penetration through tissues and biological barriers (e.g., blood-brain-barrier, BBB), provide cell and tissue specificity, stability in extra- and intracellular environment, fast cell penetration, efficient localization to subcellular compartments, and allow the targeting oligonucleotide to bind specific (on-target/off-target ratio) and stable to the intended target including full compatibility with the cellular machinery and very limited interference with non-targeted cellular processes. The design of oligonucleotide therapeutics will become even more challenging when considering patient-to-patient variation, multiple targets in the same cell and attempts for tuning levels of expression instead of completely silencing them. So far, there is no single delivery system developed yet that can fulfill all the criteria mentioned, but considerable progress has been made in solving some of the challenges. The most convenient patient compliant methods of delivery to the body e.g. pills, ointments, or inhalations, which allow patients to self-administer the drug could expand RNA therapy applications to less severe diseases or more regular interventions. This does not diminish the therapeutic relevance of RNA therapeutics since many RNA therapeutics show very long half-lives and efficacies which require very infrequent dosing which makes different routes of administration clinically very relevant. So far none of the RNA drugs on the market fulfill the criteria for self-administration, but functionalization of RNAs with lipids and other hydrophobic moieties as well as formulation with lipid nanoparticles allows for absorption through the skin or nebulization and thereby lung delivery [15,16]. The uptake of RNA formulations by cells happens most frequently through clathrin-dependent or clathrin-independent endocytosis (Figure 3). Cell penetration and endocytosis can also be mediated by cell surface receptors, e.g. GalNAc-conjugated RNAs which bind to the hepatocyte-specific asialoglycoprotein receptor (ASGPR)[17]. The receptor-mediated cell penetration is also hypothesized to be responsible for gymnotic delivery of RNAs [18], which is the prevalent way of delivery of some currently approved ON drugs (Figure 2 -‘Formulation’). After formation of intracellular vesicles, the drug enters an endosomal maturation pathway. If not fused with ER or other membranes, late endosomes will fuse with lysosomes and degrade the cargo. This is considered as the most prevalent degradation pathway of synthetic ONs. Depending on the formulation, more than 90% of all RNA cargo may be degraded this way and therefore only the remaining fraction would exert the desired biological effect[19]. In addition, the lack of fully characterized mechanisms responsible for intracellular release makes the development of more effective delivery solutions increasingly difficult. Most strategies rely on empirical screening of multiple formulations with systematic variation of its components. This significantly increases time and cost of drug development but also of the final RNA drug, which can range from $10k-$750k per year of treatment [5]. To address some of the challenges, synthetic chemists have provided wide range of novel nucleic acid building blocks with improved properties compared to natural nucleic acids such as enzymatic stability or increased binding strength and thermal stability, many of the modifications are useful for both DNA and RNA therapeutics and have been used for both. This is exemplified in the chemical substitution of RNAs at the 2’ ribose position or backbone modifications, like PS, PMO or PNA, which significantly increase stability against nucleases when compared with unmodified RNAs[20]. Unfortunately, some combinations of modifications, like 2’-O-Me, PS and conjugation of hydrophobic compounds, can have deleterious effects on pharmacodynamics and immune response[21]. Some patients show hypersensitivity reaction to biologicals and may experience inflammation exacerbation after treatment with free, conjugated or LNP formulated RNAs [22,23]. Complement activation is a frequent reaction to RNA therapeutics, therefore development of strategies to avoid immune response are crucial. A common strategy for LNPs is to conjugate polyethylene glycol polymer (PEG) to the surface of nanoparticles or addition of PEGylated lipids to LNP formulations[24]. PEG prevents opsonization and recognition by macrophages leading to higher stability, longer circulation times and lower immune response[25]. However, the use of PEG in delivery formulations has been very sucessful but is not without challenges and often associated with immunogenic responses, such as triggering the rise of anti-drug antibodies (ADAs, e.g., anti-PEG IgG and IgM) resulting in faster clearance or hypersensitivity reactions (HSRs). Another strategy is to conjugate immunomodulators e.g. regulators of complement activation that down-regulate complement response[26]. Despite the progress, some therapies require frequent administration of the RNA drug which increases the chance to develop allergic reactions. Therefore, the safety of RNA drugs and patient response needs to be monitored continuously, additionally increasing the costs of therapies. There is no doubt, that development of biologically effective and cost-effective RNA therapies remains challenging. Progress in oligonucleotide chemistry helped to establish RNA compositions and sequence designs that are stable and efficient with lasting therapeutic effects. RNA therapies, approved or in clinical trials, which use one or more of the first generation modifications (Fig 4 – Gen1), they can be classified as a foundational technology, and products primarily using sugar and backbone modifications as first-generation RNA therapeutics (Figure 4 – ‘Gen.1’), they often need to be delivered in relatively high doses of up to 60 mg/kg of body weight [27] which impacts production costs of such RNAs or RNA analogues, turning some therapies prohibitively expensive. However, major advances in large scale production of shorter RNAs has reduced the production cost aspect considerably and the major driving forces for development of higher efficacy RNA therapeutics is to reduce off-target effects and more generally to improve the toxicity and safety profile. Additional functionalities can improve efficacy further, by conjugating new functionalities (e.g. targeting ligands) or increase stability by encapsulating RNA oligonucleotides into LNPs which allows to modulate the functionality of the formulation. The indicated strategies will be described more detailed for different formulation strategies as there is a great progress in both areas, covalent conjugation and LNPs. Examples for RNA drugs employing LNPs are already on the market (Patisiran), but many more RNAs covalently conjugated with functional molecules are in clinical trials and promise further development of successful RNA based therapies. We classified covalent conjugation and LNP encapsulation as second-generation RNA therapeutics as they use modified oligonucleotides in combination with other strategies (Figure. 4 – ‘Gen.2’). Effective LNP formulations and chemical RNA modifications are crucial to design more complex delivery strategies that can address challenges related to RNA delivery. Functionalized RNAs (e.g. lipidated RNAs) in combination with LNPs allow for a mix and match strategies as well as delivery of multiple different RNAs together with immunomodulators and enhancers of cell recognition and cell penetration [28–30].

Recent research on extracellular vesicles shows a great promise of developing multifunctional biomimetic nanoparticles of native origin i.e. fully compatible with patients’ immune system. All these strategies will be classified as third generation of RNA therapeutics in the current review, implementing all previous strategies of chemical modifications and lipid encapsulation or lipid membrane anchoring of functional molecules (Fig. 4 – ‘Gen.3’).

## Covalent conjugation of functional molecules to RNA

Direct conjugation of other functional molecules to RNA allows to create multifunctional drugs, with improved biological or physico-chemical features. The focus is on major classes of molecules used to improve cell targeting (small molecules, carbohydrates, antibodies, and aptamers) or enhancement of cell penetration and intracellular distribution (peptides and hydrophobic compounds). Sometimes, conjugation provides additional benefits of stabilizing RNA oligonucleotides against nucleases or adds functionality such as allowing for receptor-mediated endocytosis and subcellular localization. There are multiple strategies to conjugate molecules to ONs that can be divided into in-line conjugation and post-synthesis conjugation. The most common method for synthesis of modified RNA ONs is based on phosphoramidite chemistry, many commercially available phosphoramidites provide convenient access to a broad range of building blocks for internal and terminal incorporation of non-natural functional groups [31–35], including chemical ‘handles’ for post synthetic amide [36], thiol [37] or click [38] coupling reactions. Alternatively, modifications can be incorporated at 3’ terminus via linker modification before ON synthesis [39,40] or at the 5’ terminus as a terminal step of the synthesis [41,42]. The post-synthesis modifications can be performed while ONs are still attached to solid support or after cleavage and deprotection i.e. in solution. Solid-phase reactions have the advantage of user-friendly procedures which often provide good purity. Due to full biocompatibility and orthogonality, Cu-assisted azide-alkyne cycloaddition (CuAAC) or Copper-free ring-strain promoted azide-alkyne cycloaddition (SPAAC) are among the most popular reactions for ON modifications allowing for fast and efficient conjugation of small molecules, carbohydrates, peptides, hydrophobic compounds (including lipids), aptamers, and antibodies [43]. Examples of various conjugation strategies of different compounds are described in the following subsections, showing the vast potential of bioconjugation in developing efficient RNA therapeutics. The selection is not exhaustive but shows the broad chemical space and underlines the breadth and depth of applications and common strategies to solve multiple challenges in RNA delivery.

### Small molecule conjugation

Small molecules are monomeric molecules which add new functionality or change biophysical properties, e.g. non-nucleotidic building blocks, receptor targeting ligands, and fluorescent probes. Fluorescent labelling is the most frequently used conjugation with a multitude of applications from fundamental molecular biology studies[44], diagnostics [45] to photodynamic therapy [46]. Although not directly involved in modulation of RNA delivery, fluorescent labels help to uncover the mechanisms and dynamics of cell penetration and subcellular distribution, including studies with single cell and single-molecule resolution[44]. The conjugation of small molecules is as well explored as to be expected from the large chemical space available, which is surprising, considering the great number of known ligands specifically binding to cell surface receptors or other proteins involved in endocytosis or cellular transport. One example is anisamide conjugation to 3’ end of ASO that showed that single group coupled through a linker provided only small changes in cell uptake and gene expression, but modifying the conjugate to a trivalent version i.e. triple linker-anisamide group bound by three-branched linker to 3’ end of ASO, showed 2-fold increase in uptake and 4-fold in gene expression[47]. The challenge with small molecule conjugation is the preservation of receptor binding specificity while making the conjugate chemically compatible with in-line or post-synthetic conjugation. Receptor binding specificity is also affected by proximity of the highly charged ON backbone and therefore extending linker length between ON and conjugate may improve recognition and internalization. Engineering linker structures allows for additional functionality as shown by Orellana et al. [48]. The group first developed folate conjugated miRNA by a copper-free ‘click’ reaction, with a ON-conjugate that provided specific *in vivo* binding to folate receptors (FRs) which are overexpressed in many cancer cell types [49]. In the next study, they added nigericin (potent activator of the NLRP3 inflammasome) via a disulphide bond to the linker region connecting folate to the 5’ terminus of the passenger strand of miRNA. After FR-mediated endocytosis, nigericin dissociated from the linker and acted as an ionophore to transport potassium across the membrane causing endosomal swelling and improved miRNA release to the cytosol[48]. Exploiting disulphide-based conjugation chemistry also enabled effective codelivery of doxorubicin (Dox) and siRNA to cancer cells[50]. The direct conjugation solves a problem of premature dissociation of Dox and unintended toxicity that was observed in non-covalent formulations of Dox and siRNA. Even though most developments are focused on targeting human cancer cells, there is also potential in targeting and modifying bacteria, especially related with human infectious diseases or the human microbiome. Direct conjugation of cobalamin (vitamin B12) to miRNA using ‘click’ chemistry allowed for efficient delivery of miRNA to *E. coli* and *S. typhimurium* and reduction of expression of target genes, without toxic effects to mammalian cells[51].

### Carbohydrate conjugation

Glycosylation of biological compounds is a natural process with a wide range of functions, from molecular recognition and interaction through cell communication to detoxification which turns synthetic carbohydrate-conjugation into a useful tool in developing diagnostic and delivery strategies. Due to the availability of well-established enzymatic and chemical methods, protein and peptide glycosylation is commonly used, but recently glycosylation methods for other molecule classes, including ONs, have been developed. D’Onofrio et al. developed phosphoramidites for conjugation of mono- and disaccharides at the 3’ or 5’ terminus of ASO during automated synthesis[52]. The G-quadruplex-forming glycosylated sequence showed antiviral activity against HIV, although addition of a bulky *tert-*butyldiphenylsilyl group at 5’ terminus significantly improved the activity, questioning the effectiveness of glycosylation itself. What is clear, is that glycosylation at both termini effectively prevents degradation by exonucleases, that require unmodified 3’ or 5’ ends and are the most abundant nucleases in cells[53]. Beyond ON protection, glycosylation can add new modalities, improving organ distribution, cell specificity, membrane penetration and endosomal release of ONs. The specific recognition of glycosylated ONs by surface receptors was employed in studies of Zhu and Mahato[54]. Targeting of different liver cell types, hepatocytes, or hepatic stellate cells, was possible by conjugation of galactose or mannose-6-phosphate (M6P) via an acid-labile PEG linker to the 3’ terminus of the siRNA’s passenger strand. Galactose is recognized by asialoglycoprotein receptor (ASGPR) on the surface of hepatocytes and M6P by M6P/insulin-like growth factor II-receptor (M6P/IGF2R) on the surface of hepatic stellate cells, in both cases improving silencing of the reporter gene by 40% in *in vitro* studies. Interestingly, addition of cationic lipids improved silencing to 98%, pointing to inefficient endosomal escape when using glycosylated siRNA alone[54]. Recently, targeting of ASGPR became widely explored as efficient way of delivering ONs to hepatocytes. Research on this receptor family helped to establish its preference for terminal N-acetyl galactosamine (GalNAc) over galactose (from 2 to 60 fold) and showed significant improvement of ligand affinity as a function of its multivalency, corresponding to the oligomeric state of ASGPR[55]. Comparison of terminal triantennary vs triple monovalent GalNAc conjugated at different locations of the siRNA showed very small differences in cell uptake and maintained silencing as long as GalNAc was conjugated as triple cluster (no extra nucleotides between) and close to 3’ or 5’ terminus [56]. The terminal triantennary GalNAc is the most successful design so far, and shows good hepatocyte incorporation compared to other designs e.g. with modified linker regions, but provides multiple chemical strategies for conjugation to ONs [57,58]. The combination of sugar or phosphor backbone modified ONs together with GalNAc conjugation dramatically improve the performance of silencing e.g. incorporating S-2-O-Et-2,4-bridged nucleic acids (ENA) with improved silencing by 61-fold or combination of LNA and phosphorothioate by at least 4-fold [17,59]. Compared with hydrophobic conjugates such as cholesterol or tocopherol, triantennary GalNAc also showed very high efficiency and specificity, although the exclusive accumulation in hepatocytes may also be considered as a drawback[60]. Nevertheless, detailed studies of GalNAc conjugated siRNA’s metabolism on animals (including monkeys) proved very favourable in respect to the safety profile in addition to excellent pharmacodynamics[61]. The discovery of enzymatic reactions involved in metabolism of triantennary GalNAc conjugates allowed for detailed phenotyping during treatment. Additionally, recurrent confirmations of low toxicity when administered in high doses with excretion of >90% in 48 h, helped to progress RNA drug development from research to clinical trials. In phase 1/2a clinical studies, administration of GalNAc conjugated ASO targeting apolipoprotein C-III (apoC-III) mRNA, showed clear dose dependence, up to 92% reduction in apoC-III protein levels and up to 77% reduction in triglyceride levels, which is very promising for patients with hypertriglyceridaemia or coronary heart disease[62]. In another phase 2 study, GalNAc conjugated ASOs against angiopoietin-like 3 protein (ANGPTL3) mRNA, reduced triglycerides up to 53% and total cholesterol up to 19%, with overall positive change in lipid and lipoprotein profile, showing another promising strategy in cardiovascular risk reduction[63].

### Hydrophobic molecule conjugation

Covalent conjugation of lipids and other hydrophobic molecules to RNA significantly changes the biophysical behaviour in aqueous environment by inducing potential self-aggregation depending on sequence design or partitioning to the surface of lipid membranes[64]. RNA lipidation is advantageous for LNP formulation design especially in the in context of complex delivery systems based on liposomes or extracellular vesicles. Hydrophobic molecules may also improve protein interactions (e.g. albumin), including receptor-specific interactions, or may add extra functionalities, some lipids provide different biodistribution with accumulation in other tissues than liver, higher serum stability or cell specificity. Size and hydrophobicity (amphiphilicity) of lipids impact the synthesis and purification of lipidated ONs (LiNAs) and make especially multiple incorporations and increasing chain length (≥ C14 carbon length) more challenging. The in-line incorporation is possible if compatible phosphoramidites or modified solid supports are available, which most often requires custom made building blocks[31]. Even if modified building blocks are available, the efficiency of automated ON synthesis is influenced by the position and number of incorporations, the lipid size and synthetic challenges caused by increased hydrophobicity of the solid support surface by the growing lipidated ON (LiNA)[40]. Alternatively, post-synthesis conjugation can be performed using ‘click’ chemistry[65], terminal amino modification [66,67], maleimides [29] or reducible groups [68]. Using any of the strategies, losses of product often occur during purification, where the amphipathic nature of LiNAs may cause aggregation and result in difficult chromatographic separation especially on commonly used reversed-phase chromatographic phase (e.g. C18, C8). Soutschek et al. showed that conjugation of cholesterol (Chol) at 3’ terminus of siRNA, not only improved silencing efficiency, but in absence of additional transfection reagents, Chol-siRNA bound to human serum albumin (HSB) and improved *in vivo* stability 16-fold and was significantly accumulated in liver, heart, kidney, and lungs[20]. Chol-siRNA showed also very high loading efficiency (_~_99%) when prepared with reconstituted high-density lipoproteins (rHDL), that delivered siRNA to hepatocytes and reduced Pokemon and Bcl-2 protein expression in *in vitro* and *in vivo* experiments[68]. Incorporation of cleavable linkers such as hydrazone or disulphide between Chol and siRNA allowed to create a carrier-free, pH-responsive human mitochondria transfection system, that efficiently targeted a point mutation in the *ND5* gene[69]. The hydrazone linker was more effective in releasing siRNA, due to faster kinetics in acidic environment, present in endosomes, and high stability in neutral or basic pH, present in extracellular environments. Comparison of cholesterol and octadecanol conjugated siRNAs showed improvement in cell penetration and antiviral activity against hepatitis C virus with the more hydrophobic (octadecanol) group[65]. Similarly, comparison of non-conjugated, Chol-conjugated, lauric (C12)- or palmitic (C16) acid-conjugated siRNAs showed gradual improvement with increasing hydrophobicity C16> C12> Chol>None, with a significant preference of 5’- over 3’-terminus conjugation [66]. There is no consensus about positioning of the lipid modifications as it might depend on the overall design of the required RNA. Kubo et al., showed that a two-sided RNA overhang design for Dicer-substrate siRNA (DsiRNA) leads to higher *in vitro* activity when conjugated with C16 at 5’-terminus, but changing the design to an asymmetric, 3’ blunt ended DsiRNA, showed better silencing of 3’ conjugated C16, although it’s not clear if that is because of an improved DsiRNA conformation or Dicer protein preference[70]. Direct conjugation of phosphatidyl lipids at 5’-terminus of ASOs showed similar relationship as for fatty acids i.e. longer lipid chain length with improved silencing[71]. Potential for long carbon chains obstructing proper interaction of siRNA with the target can be alleviated by using reducible linkers e.g. 2-pyridyl disulphide, that are stable in extracellular environment but release siRNA from the lipid inside the cell [72]. That is what Musaccio et al. have used, creating siRNA-S-S-phosphothioethanol (siRNA-PE), that spontaneously formed micelles and allowed to mix with PEG-PE for non-toxic delivery[72]. Spontaneous partitioning of LiNAs into lipid membranes was also applied for delivery of G4-decoy against *KRAS* gene expression regulator in pancreatic cancer cell line (Panc-1) in *in vitro* studies [73,74]. Cogoi et al. used lipid mimicking phosphoramidite with two palmityl chains incorporated at 4^th^ position from 3ʹterminus, that allowed for efficient binding of lipidated G4-decoy ON to the surface of palmitoyl-oleyl-phosphatidylcholine (POPC) liposomes[73]. This formulation allowed to add lipidated cell-penetrating peptides (CPPs) to the surface of the liposomes and effectively deliver ONs to pancreatic cancer cells[73]. Lipidation of ONs allows for anchoring them to the surface of cells e.g. cancer cells, where ONs mimicking pathogen signatures induce immune response against the cells, this has been exemplified by Liu et al. using lipidated immunostimulatory CpG-oligonucleotides to trigger an immunostimulatory cascade (which could be extended to immunostimulatory lipidated dsRNA to mimic molecular signatures of pathogens such as viruses or bacteria)[41]. This opens a path to localized, cancer immunotherapy and anti-cancer vaccines. Additional modifications of conjugated lipids e.g. by terminal lipid phosphorylation, allows to anchor ONs to cancer cells only if they overexpress membrane exposed alkaline phosphatase, a common cancer cell marker[75]. Lipidation was employed to silence genes in hard-to-transfect colorectal cancer cells (HT-29) [76]. Kubo et al. tested in *in vitro* experiments saturated and unsaturated fatty acids and found that C16 and C18 carbon length in *cis*-form are optimal for transformation with lipofectamine, but without lipofectamine only longer carbon chain conjugates, C22, eicosapentaenoic acid (EPA) and Docosahexaenoic acid (DHA), showed significant activity[76]. Targeting other challenging cells, like neuronal or immune cells, can be achieved by conjugation with receptor specific hydrophobic compounds e.g. anandamide[77]. When lipid-siRNA conjugates are injected *in vivo* they quickly bind to serum albumin which improves RNA stability and bioavailability. Diacyl lipid (C18) conjugated to siRNA showed almost 99% uptake in the mice tumour site and over 40:1, tumour: liver accumulation ratio, which was 1,6 fold better uptake and 13 fold better accumulation ratio than unmodified siRNA compared with an *in vivo* commercial transfection formulation [78]. ‘Hitchhiking’ albumin for distribution of floxuridine, a chemotherapeutic, was employed by incorporated to homomeric ON, conjugated with di-stearyl group (C18)[79]. In solution, ON-lipid conjugate spontaneously formed micelles, but after *in vivo* injection it bound to serum albumin and was effectively distributed to cancer cells. Jin et al. have exemplified this by synthesis of lipidated single stranded floxuridine homomers which attach to Albumin and are subsequently transported and accumulated in cancer cells and generate free floxuridine upon lysosomal degradation which interferes with DNA synthesis and inhibits cell proliferation [79]. That shows potential for creation of multifunctional drugs combining small molecule and ON modalities. Delivery of RNAs to other organs than liver is still challenging, but lipid conjugation represents a promising strategy. Biscans et al. tested a library of lipids conjugated to siRNA and showed differential accumulation for different lipids, e.g., lithocholic acid (LA), phosphocholine (PC)-EPA and PC-DHA accumulated mostly in kidneys but not in liver, or docosanoic acid (DCA) and PC-DCA accumulated mostly in heart, lungs and muscles[80]. Godinho et al. showed, that conjugation of PC-DHA with siRNA allows successful delivery across BBB and distribution in mice brain using intracarotid injection and mild osmotic pressure[81]. In another study, Biscans et al. used the same library of lipid-siRNAs and showed efficient loading of conjugates to the surface of extracellular vesicles (EVs) and successful silencing of *Huntingtin (Htt)* mRNA[82]. Alpha-tocopheryl and PC-α-tocopheryl showed the best loading efficiency which correlated well with the low zeta potential of siRNA-EV complexes and their high efficiency in gene silencing[82]. The tissue distribution correlates well with endothelial transport to interstitium, and caveolin-dependent or FcRn-mediated transcytosis[83].

### Peptide conjugation

Well-established peptide synthesis strategies and a large number of commercially available natural and non-natural peptide building blocks make peptides attractive for ON-conjugation. Specific peptides can modulate immune response, improve RNA stability, cell targeting and specificity (peptidic ligands), cell penetration (CPPs) and endosomal release (cationic peptides). Peptide synthesis is also compatible with alternative backbone chemistries i.e. PMOs and PNAs, that significantly alter the biophysical properties of oligonucleotides and have already shown to be safe in some clinical applications [84,85]. CPPs are among the most commonly used peptide conjugates but are often associated with toxicity presumably based on their sequence design with often multiple positive charges. Despite low toxicity, great stability and sequence specificity, PMOs and PNAs show very limited bioavailability, therefore conjugation to CPPs can dramatically improve their bioavailability [86,87]. Polycationic peptides, like polymeric arginine (R6-R12) or Tat (derived from the transactivator of transcription of HIV-1), are conjugated at 5’- or 3’-terminus of ONs and drive cell internalization, most frequently, via endocytotic pathway[88]. Unfortunately, peptide conjugates are often not efficiently released from endosomes and end up in the lysosomal degradation pathway. Early research to alleviate this problem used endosomolytic agents, like chloroquine or high calcium concentration, but this was only applicable in *in vitro* experiments[87]. Conjugating different peptides or combinations of cationic peptides with endosomolytic peptides, like fusogenic peptides derived from haemagglutinin envelope protein (HA)[89] or R6-Penetratin [90], improves endosomal release and overall silencing efficiency. Addition of 6-aminohexanoic acid (Ahx or X) as a spacer between arginines (RXR)_4_ significantly improved PMOs’ delivery *in vitro* and measured activity, in the absence of endosomolytic agents [91]. In similar studies, comparison of Ahx or β-alanine (B) incorporation to octa-arginine (RXR)_4_ vs (RBR)_4_ showed improved serum stability and higher activity of the β-alanine version peptide [92]. Addition of β-alanine after (RXR)_4_ showed even better extra- and intracellular stability with superior PMOs antisense activity [92]. The conformation of the conjugated peptide might be affected by the proximity to the ON, therefore the use of reducible linkers, disulphide or thioether, is needed to maintain the fusogenic and endosomolytic activity[93]. Despite available chemical methods to efficiently conjugate more than one peptide to RNA e.g. using thiazolidine, oxime or hydrazine linkages, the silencing activity might not be improved, showing the design challenge in adding more than one functionality to ONs by bioconjugation [94]. The first approved ON therapeutic, Fomivirsen, was targeted against cytomegalovirus’ mRNA and provided proof-of-concept for further development of ON-based antiviral therapies[95]. Conjugation of arginine rich peptide (RXR)_4_XB to 5’-terminus of PMOs targeting conserved *cis*-regulatory elements of flavivirus’ RNAs (mosquito-borne and West Nile viruses), showed effective inhibition of viral protein expression and viral RNA replication [96]. Similarly, conjugation of various arginine rich peptides to PMOs allowed for significant titre reduction and viral suppression of other viral infections e.g. dengue[97], measles [98], infectious haematopoietic necrosis [99], influenza [100]. Years of development of nucleoside/nucleotide based antiviral therapeutics allowed for fast implementation of this knowledge to the major public health crisis in recent history by providing a directly acting antiviral as complementary tool to mRNA based vaccines for SARS-CoV-2 pandemic[101]. (RXR)_4_ conjugated to PMO against 5’ terminal or leader-TRS region reduced >95% of viral titres 12–48 h after cell infection [101]. Implementation of automated single-shot fast-flow synthesis was to optimize and produce (RXRRBR)_2_ conjugated PNAs, which also reduce >95% of SARS-CoV-2 titres, promise to quickly develop and deliver viral-strain-specific drug to the market, *in vivo* antiviral efficacy was demonstrated by PPMO against several respiratory viruses in previous studies [102]. Beyond viral infections, Tat conjugation to PNA, targeting *gyr*A gene, significantly inhibit *S. pyogenes* cell growth, and pioneered a promising strategy of fighting multidrug resistant bacterial strains[103]. Targeting specific organs, cells and subcellular compartments can be achieved by conjugation of ONs to specific peptides. Frequently used cyclo(RGD) peptide, or modified version cyclo(RGDfK)X, in 1:1, 2:1 or 3:1 peptide:ON conjugation ratio, can very selectively bind to αvβ3 receptors, overexpressed on the surface of various cancer cells [104]. Cen et al. showed slowed glioblastoma growth after IV injection of cyclo(RGDfK)X conjugated siRNA targeting phosphatidylinositol-4,5-bisphosphate 3-kinase catalytic subunit beta mRNA in mice brain [105]. Conjugation of siRNA with signal peptide for *trans*-membrane transport of bacterial protein toxins allowed for efficient siRNA release from the caveosomal pathway and targeting of the ER-specific perinuclear site in a model endothelial cell line (ECV304) [106]. Similarly, conjugation of mitochondrial targeting peptide to PNA allowed uptake of the conjugate to mitochondria and modulation of protein expression[107]. Using D-amino acid analogues of insulin-like growth factor I (IGF1) conjugated to PNA showed improved uptake only to cells overexpressing IGF1 surface receptor[108]. Using another peptidic ligand, neurotensin (NT), that has high affinity to sortilin receptors expressed in the CNS, Nikan et al. showed high specificity and uptake efficiency to sortilin expressing cells for modified RNA and PMO ASOs correcting splicing of pre-mRNA[109]. Currently available drugs on the market for treating Duchenne muscular dystrophy (DMD) are effective but new developments in conjugation technology show promise of further improvements. The octaarginine (RXR)_4_ conjugate with PNA shows strong *mdx* exon skipping efficiency in myotube cells of DMD model mice [110]. Comparison of similar cell penetrating peptides (R)_8_XB and (RXRRBR)_2_XB showed high accumulation in liver or in quadriceps and heart, respectively, in DMD model mice [111]. Another variant of arginine rich CPP with X and B (RXRRBRRXRYQFLI(RXRB)_2_) called PMO internalizing peptide 6a (Pip6a) conjugated to splice-switching PMO, targeted exon 7 of survival motor neuron 2 (*SMN2*) pre-mRNA and showed significant distribution to the CNS after systemic administration, and resulted in 38-fold extended life expectancy in a spinal muscular atrophy (SMA) mice model [112]. An even simpler CPP version, (RXRRBR)_2_XB, can also be delivered as PMOs across BBB and substantially increases SMN protein levels, alleviating major SMA symptoms in mice [113]. Covalent conjugation of Protein Transduction Domain (PFVYLI) to *in vitro* transcribed mRNA allowed SCO2 protein complementation in *SCO2/COX*-deficient primary fibroblast and β-globin production in cells from β-thalassaemic patients[114].

### Protein, antibody conjugation

Extending peptide conjugation to larger molecules, protein conjugation adds new modalities (functionalities) to RNA, expands available libraries of antibodies (Abs) and significantly improves cell selectivity. An interesting example is redirecting adenosine deaminase acting on RNA (ADAR) protein to a new target-of-choice, by substitution of N-terminal, dsRNA-binding domains of the ADAR with SNAP-tag domain, that chemo-selectively forms a covalent bond with 5’-*O*-benzylguanine-modified guide RNA (gRNA)[115]. New gRNA-SNAP-tag-deaminase conjugates can effectively edit adenosine-to-inosine (A-to-I) in target RNA, directed by an A:C mismatch in the middle of the gRNA [115]. The specificity of monoclonal antibodies allows targeting of challenging diseases like leukaemia. Satake et al., used a DBCO-modified ASO against a critical transcription factor in precursor B-cell (preB) acute lymphoblastic leukaemia (ALL) to react with an azide modified anti-CD22 Ab, and showed preferential cytotoxicity in preB cells and doubling of the life expectancy in ALL model mice[116]. In hard to treat rheumatoid arthritis (RA) patients, conjugation of siRNA against C5 complement’s component with C5a receptor 1 (C5aR1)-specific Ab showed 83% reduction in RA-related symptoms compared to only 19% when siRNA and Ab were delivered unconjugated [117]. The possibility to introduce precisely positioned cysteine to antibodies’ heavy chain allowed covalent coupling of two siRNA per Ab[118]. Introduction of 3’ amine in siRNA’s passenger strands and conjugation via reducible N-succinimidyl4-(2-pyridyldithio)butyrate (SPDB) or non-reducible succinimidyl-4-[N-maleimidomethyl]cyclohexane-1-carboxylate) (SMCC) NHS (N-hydroxysuccinimide) linkers created a flexible, precise and biorthogonal conjugation platform for straightforward generation of Ab-siRNA conjugate (ARC) libraries, that were able to deliver conjugates to tumour cells *in vivo* [118]. The only major limitation was inefficient endosomal escape of these ARCs. Conjugation via cationic gelatin linker of KRAS_G12C_-specific siRNA and Cetuximab, allowed efficient KRAS-mutant downregulation in lung cancer cells and their sensitization to the small molecule chemotherapeutic, Gefitinib[119].

### Aptamer conjugation

Despite functional similarities between antibodies and aptamers [120–122], the latter show advantages with their ability to create multivalent, multimodal formulations with remarkably high binding affinity and specificity using relatively simple and inexpensive methods. Straightforward preparation of biotinylated aptamers and siRNAs allowed the preparation of multi-aptamer:multi-siRNA:Streptavidin conjugates that preferentially bind to prostate-specific membrane antigen (PSMA) expressing cells and reduced the production of target proteins (lamin-A/C or GAPDH) [123]. This formulation performed as good as a formulation with commercial transfection reagents. By systematic evolution of ligands through exponential enrichment (SELEX) it was possible to find aptamers with sub-μM affinity to serum albumin which protected aptamer-siRNA chimeras and increased circulation half-life by about 60%[124]. Yu et al. showed that a preparation of a tri-functional aptamer-siRNA chimera, with two, terminal aptamers targeting human epidermal growth factor receptor 2 (HER2) and 3 (HER3) and siRNA targeting *EGFR* mRNA in HER2^+^ breast cancer cells can specifically and efficiently inhibit breast cancer growth in a mice model system. The design used is a noncovalent approach where both HER2 and HER3 targeting aptamers are coupled by hybridization of two complementary 19mer sense strands and anti-sense EGFR siRNA sequences, both linked to the respective aptamers[125]. They demonstrated a promising solution to compensatory mechanism when only one HER receptor is blocked resulting in resistance to classical therapies[125]. Crosslinking of multiple antisense strands in line via reducible dithio-bis-maleimidoethane, followed by hybridization of the sense strand with anti-mucin 1 (MUC1) aptamer and intercalating doxorubicin (Dox) to the stem of the aptamer showed specific targeting to human breast cancer MCF-7 cells, silencing of antiapoptotic *BCL2* mRNA and inhibition of cell proliferation[126].

## Lipid nanoparticles (LNP

Lipid-based nanoparticles are among the most well established nanoparticle systems applied to delivery of RNA[30]. By encapsulation of RNA, LNPs protect cargo from extracellular nucleases allowing safe delivery of unmodified ONs to cells. Complexation of ONs with cationic lipids, like 1,2-di-O-octadecenyl-3-trimethylammonium-propane (DOTMA) or 1,2-dioleoyl-3-trimethylammonium-propane (DOTAP), neutralize charges, condense long ON chains, and improve encapsulation when mixed with zwitterionic lipids, such as 1,2-dioleoyl-*sn*-3-phosphoethanolamine (DOPE) and dioleoyl phosphatidylcholine (DOPC). Delivery of ONs complexed only by DOTMA, or other cationic lipids, is known as lipofection, and is the most popular *in vitro* transformation method[127]. Unfortunately, most lipofection reagents are not suitable for *in vivo* delivery due to high cytotoxicity making liposomal formulations more suitable for therapeutic applications[128]. In more recent LNP formulations, cationic lipids are substituted by ionizable lipids, such as analogues of 1,2-dioleyloxy-N,N-dimethyl-3-aminopropane (DODMA), which are positively charged during complexation, neutral once encapsulated, and again positively charged in endosomes, allowing to interact with endogenous, negatively charged lipids and promote cargo release [129]. In *in vivo* applications, this simple formulation can elicit inflammatory response and if unresolved can create entrained allergic reaction or even death[130]. Therefore, addition of PEGylated lipids to LNP formulations became popular resulting in lower opsonization and increased circulation time. At the same time, PEG coated LNPs show much lower cellular uptake and hindered endosomal escape, creating the so called ‘PEG dilemma’, where too little PEGylate lipids lowers circulation time but too much lowers efficacy [131]. One of the solutions is to use pH-sensitive lipids which cleave the PEG group after endocytosis improving cargo release, although that still doesn’t improve first step, of cellular uptake[132]. PEGylated lipids can also induce ‘accelerated blood release’ phenomena, were repeated administration of PEGylated liposomes lowers its circulation time and can create innate immune response known as complement activation-related pseudo-allergy (CARPA)[133]. When LNPs are bigger than 150 nm in diameter they will rarely leave blood vessel’s capillaries to other organs and are cleared mostly via the reticuloendothelial system in liver and spleen[134]. Even if these LNPs will not be degraded by macrophages they will mostly accumulate in liver cells. To target other organs, Cheng et al., developed a library of permanently charged and ionizable lipids (SORT), defined by their optimal ratio in LNP formulations, and showed that changing the ratio of DOTAP, DODAP or 1,2-dioleoyl-sn-glycero-3-phosphate (18 PA) to the remaining lipids lead to preferential accumulation of LNPs in spleen, lungs or liver [135]. Similarly, Ramishetti et al., screened a library of novel ionizable amino lipids for improved delivery to leukocytes, which are considered particularly challenging to transfect[136]. They found two promising candidates, that in LNP’s composition of lipid:Chol:1,2-distearoyl-*sn*-glycero-3-phosphocholine (DSPC):PEGylated lipid (50:38.5:10:1.5), showed a very low polydispersity index (PD) <0.1 with an average diameter of 50 nm, which are very desirable characteristics for *in vivo* applications. These LNPs with Lipid10 (lipid with a *N*-methyl-piperazine head group) showed high accumulation in spleen, and after LNP surface modification with anti-integrin β_7_ mAb using intravenous administration to mice, were able to modulate CD45 expression exclusively in primary lymphocytes [136]. Exploiting the self-assembly characteristics of lipids and lipidated molecules, adding more functionalities to LNP formulations can be achieved by simple addition of lipidated functional entities during or after LNP preparation. Ferino et al. used palmityl-oleyl-phosphatidylcholine (POPC) liposomes and functionalized their surface with lipidated miRNA (miR216b), complementary to protooncogenic KRAS_G12D_ mutant sequence (the specificity or level of off-target effects are not reported), and lipidated Tat peptide as CPP, improving cell penetration to pancreatic ductal adenocarcinoma (PDAC) cells[137]. Incubation of this formulation with PDAC cell lines in *in vitro* studies showed up to 70% of reduction in mutated KRAS protein production. Conjugation of different functionalities to PEG-DOPE allowed Pan et al. to create multifunctional micellar LNPs[138]. They created G4-polyamidoamine dendrimer (PAMAM)-PEG-DOPE that efficiently bind siRNA, and 2C5 mAb-PEG-DOPE that specifically binds to cancer cells. Adding Dox during micelle formation created a multifunctional formulation, with a specific antibody improving cancer cell recognition and penetration, siRNA reducing P-gp expression and sensitizing tumour cells to doxorubicin activity[138]. Few LNP based RNA formulations have progressed to clinical trials and or finally succeeded to become FDA approved RNA therapeutics. The development of successful formulations for delivery of siRNA inhibiting synthesis of transthyretin (TTR) in hepatocytes took at least two decades but became the first approved liposomal siRNA therapy for transthyretin-mediated amyloidosis, Patisiran (marketed as Onpattro) [139]. Crucial improvements were done by systematic engineering and testing of ionizable lipids[140]. First, by developing stable nucleic acid lipid particles (SNALPs) characterized by very efficient RNA encapsulation, small and homogenous size, and >90% effective, long-lasting gene silencing in non-human primates[141]. Later, the structure of ionizable lipids to maintain high RNA encapsulation efficiency and bilayer conformation before cell internalization has been optimized to maximize interaction with endosomal anionic lipids and subsequently promotion of effective membrane disruption at the same time [142]. LNPs containing heptatriaconta-6,9,28,31-tetraen-19-yl-4-(dimethylamino) butanoate (DLin-MC3-DMA) showed great pH responsiveness, size tunability, very low toxicity, and immunogenicity, and was used in commercial formulations [143]. An ionizable lipid selection was also necessary for successful development of mRNA-based SARS-CoV-2 vaccines from Pfizer and Moderna. Because vaccines are usually administered intramuscularly (IM), formulations optimized for intravenous (IV) administration with DLin-MC3-DMA were showing moderate local and systemic adverse effects in a number of preclinical and clinical experiments[144]. Scientists at Moderna, by screening libraries of ionizable amino lipids, found SM-102 (heptadecan-9-yl-8-((2-hydroxyethyl)(6-oxo-6-(undecyloxy)-hexyl)-amino)-octanoate) that supported efficient mRNA delivery and expression, with desirable high immunogenicity and much lower inflammatory response than DLin-MC3-DMA was induced after IM administration [145]. During the worldwide distribution of COVID-19 vaccines, low and ultralow storage temperatures became a serious limitation and presented a major expense. It is speculated, that the biggest role of the low mRNA vaccine stability at higher temperatures is not LNP aggregation or degradation but changes to the encapsulated mRNA, either by phosphodiester bond hydrolysis or nucleobase oxidation[146].

## Extracellular vesicles

The natural role of extracellular vesicles (EVs) as ‘transporters’ of biological material between cells, tissues, and organs represents one way of long-distance communication, suggests EVs as very promising vehicles for therapeutic applications. They have very diverse compositions dependent on the cellular origin of the vesicles, which includes DNA, RNA, membrane and soluble proteins, lipids, carbohydrates, and other small molecules and have shown to be able to cross many biological barriers including the blood-brain barrier. Many of these molecules are presented at the surface and can contribute to unspecific or specific cell targeting. This abundance of potential ligand-receptor interactions explains the higher efficacy of EVs internalization compared to synthetic LNPs. The outstanding challenge for therapeutic application is efficient loading with synthetic cargo, such as siRNA or ASO. EVs are characterized by surprising mechanical and chemical stability after isolation but that also limits potential methods for incorporating external molecules[8]. Electroporation is a popular and common method for cell transfection which is also used for loading of EVs. Efficiency of ON transfer to the lumen of EVs depends on the size and structure of the ON, with improved yields using linear molecules <1000nt rather than bigger and circular, but also on the size of EVs, with vesicles <50 nm in diameter being much more challenging than >80 nm[147]. Unfortunately, loaded EVs after electroporation often showed unsatisfactory levels of activity, potentially due to altered membrane composition or membrane protein denaturation caused by transient pore formation[148]. Mild conditions for loading of EVs has been achieved by targeting active molecules to their surface. O’Loughlin et al. showed that Chol-conjugated siRNA incubated with EVs for 1 h at 37°C resulted in generally higher and concentration-dependent silencing of target mRNA, human antigen R (*HuR*), in cancer cells when compared to gymnotic delivery[149]. Similarly loaded Chol-siRNA against *Huntingtin* (*Htt*) mRNA showed greatly improved *in vivo* distribution of EVs in mice brain and significant, up to 35% reduction in *Htt* mRNA[150]. An interesting method presented by Evers et al. based on coextrusion of EVs and liposomes previously loaded with siRNA created hybrid vesicles that maintained cell origin characteristics and specific recognition features but with improved cargo delivery and gene silencing when compared to initial LNPs[151]. Instead of external loading, EVs can be loaded and engineered before formation, by engineering the cells they are coming from. Exploiting significant enrichment of miR-451 miRNA in EVs from many cell-types as shown by Reshke et al. who engineered the corresponding pre-microRNA region to produce siRNA targeting GFP, tetracycline repressor protein or SOD1, and showed 7000-fold enrichment in loaded EVs and >10-fold improvement in target mRNA silencing in transgenic mice [152]. The results suggest this to be a promising strategy to significantly reduce therapeutic dosage while maintaining the desired level of activity. Considering the increased interest in multimodal therapies for cancer, it is desirable to establish efficient preparation methods for multifunctional EVs. Jhan et al. used a combination of electroporation, to internalize siRNA to EVs’ lumen, and layer-by-layer deposition of polyelectrolyte polymers at the surface of EVs, with second layer complexed doxorubicin (Dox)[153]. This formulation penetrated cancer cells better than a commercial lipofectamine reagent and showed efficient gene silencing in combination with Dox induced apoptosis. The application of CPPs to load multiple siRNAs into EVs is not obvious but shown to be possible, and it suggests active endocytotic processes in EVs or alternative mechanisms of internalization mediated by Tat peptides[154]. Diao et al. used a fusion protein consisting of three Tat peptides and a double-strand RNA binding domain (3TD) to complex three different siRNA targeting cancer marker genes, *FLOH1, NKX3, DHRS7*, and showed more than 3-fold improvement of internalization over gymnotic delivery and desired activity against all targets. Major challenges for practical therapeutic applications of EVs remain, despite their potential of as delivery vehicles. The challenges comprise isolation, upscaling, batch-to-batch variability, shelf live-related stability and the impurity profile depending on the biological source and purification techniques used. Nevertheless, their unique composition, ability to cross biological barriers and current improvements in techniques for loading and generation of EV-hybrid particles may well prove important for future EV-based delivery approaches and enable personalized formulations if patient isolated EVs are used for more specific applications.

## Polymer particles and metal-organic frameworks

In comparison to biomimetic LNPs and EVs, using polymeric materials allows to create highly controlled and diverse structures and shapes of nanoparticles. They range from spherical micelles, polyplexes and lipoplexes to cubes, rods, microneedles, and hydrogels. The functionality of formulation is mainly guided by polymer’s characteristics or by combination of various polymers, which allows to precisely finetune the functions[155]. To speed up selection of polymers for delivery of RNAs to specific tissues, it is possible to apply high-throughput polymer synthesis combined with high-throughput formulation and cell-based screening methods[156]. The most frequently used polymers are based on polysaccharides, like chitin or chitosan, poly(amino acids), like poly(L-lysine), polyamines, like poly(ethyleneimine), polyesters, like poly(lacto-co-glycolic acid) or poly(β-amino ester), and polyamidoamines, like poly(amido-butanol) [157]. ONs are mostly complexed via ionic interactions, where the polymer has some positively charged moieties, but ONs can be also entrapped in solid or porous particles where other interactions are dominant. Many of the polymers have a high density of positive charges which makes them highly effective in condensing nucleic acids and releasing ONs from the endosomes but also show physiological toxicity limiting applications to mostly *in vitro* experiments[157]. Limiting the amount of (positively) charged polymers in combination with neutral polymers or biopolymers, like lipids or peptides is a common strategy to reduce toxicity[158]. Alternative strategies involve modification of polymers with PEG or by glycosylation, where sugar moieties function as specific receptor ligand[159].

Metal-organic frameworks (MOFs) show similarities with polymeric particles by encapsulating or condensing ONs. Supramolecular, organic ligands coordinate metal ions forming porous materials that can trap ONs during formation or complex them afterwards. Similarly to polymeric nanoparticles they can form well defined structures with high loading capacity[160]. Their shortcomings of low cell specificity can be improved by coating with natural cell membranes, as shown by Zhuang et al[161]. They used platelet cell membranes for coating zeolitic imidazolate framework-8 (ZIF-8) MOFs loaded with siRNA against surviving mRNA or marker fluorescent gene. In *in vivo* studies they observed significant accumulation of MOFs in the tumour and reduction of fluorescence or slowdown of tumour growth[161]. Another hybrid system successfully delivered MOF-encapsulated siRNA via oral delivery, where anti-TNFα siRNA was incorporated to zinc based MOFs and then covered by sodium alginate, creating formulations that survived in low pH of stomach and small intestine, and significantly reduced colon inflammation in an ulcerative colitis mice model[162].

## Conclusion

The idea to use RNAs as therapeutic agents and target RNAs for therapeutic purposes emerged soon after their discovery but it took decades of research and development to bring the first ON-based drugs to the market[3]. In this review, we illustrated, through selected examples, major challenges that helped to progress RNA therapies from lab bench to clinics, namely stability of RNA linked to new chemistries developed over decades, efficient delivery to specific cell types, and intracellular unloading by receptor specific ligands or optimized biodistribution for different organs. As the current understanding of RNA regulation is still incomplete and adjusted based on new discoveries, very careful sequence design and experimental verification is needed to significantly reduce off-target effects. Stability of RNAs in nonencapsulated systems is typically provided by incorporating nucleotide modifications and is applied to most currently approved therapies based on small RNAs. The problem of RNA stability after encapsulation was specifically addressed, assuming physical separation of the ONs from external environment is sufficient, although it became apparent with large scale rollout of COVID-19 mRNA vaccines that RNA stability (chemically and shelf stability) in different formulations is important [146]. Incorporation of modified nucleobases, like *N*-1-methyl-pseudouridine instead of uridine, improved mRNA conformational stability and translation efficiency but did not address susceptibility to reactive oxygen species (ROS) that can act on 2ʹOH group and cause ON chain breaks. Addition of ROS scavengers into LNPs or use of modified lipids at the inner leaflet can alleviate the problem. Alternatively, developing strategies with polymerases that can site-specifically incorporate 2’-modified nucleotides also yield improved stability[163]. RNA delivery systems dramatically evolved in the last two decades from gymnotic delivery to multimodal, environment-responsive complex formulations. Current focus in research is devided between the development of more tissue-specific and efficient ON-conjugates based for less complex classical designs and further development of multifunctional nanoparticle-based formulations, including biomimetic EV-hybrid particle formulations which may allow future patient specific delivery systems. The most recent developments enable selective targeting of different organs and tissues, stabilization in extra- and intracellular environments, fast endosomal escape, and high bioactivity, so we expect improved RNA drug approval rates resulting mainly from improved formulations and delivery approaches on the market. However, it is surprisingly difficult to find examples of formulations that have more than two to three functions combined e.g. siRNA-directed silencing, Ab-directed cell selectivity and chemotherapeutic induced apoptosis. This can be explained by technical challenges to chemically conjugate multiple functionalities from different molecules into one ON-conjugate and maintain their full activity and useful pharmacokinetics. Another noteworthy aspect is the ratio relative between different functionalities, since most bioconjugation schemes will lead to 1:1 or similar ratios whereas ratios of > 1:10 are very difficult to realize and become even more complex with current bioconjugation techniques if more than two functionalities must be incorporated into one bioconjugate with different ratios between the individual functionalities. It is important to note, that the drug development process becomes considerably more challenging when the pharmacokinetics for different bioconjugate constructs must be re-evaluated for each construct in addition to the serious chemical challenges during synthesis and upscaling of more complex constructs with multiple functionalities. In contrast, addition and evaluation of new functionalities to LNPs can be done by straightforward mixing of different lipidated molecules at different concentrations e.g. lipidated RNAs and peptides [137] or other hydrophobic entities, without the need for covalent connections, which will non-covalently anchor to the surface of lipid bilayers driven by hydrophobic forces. Based on advances in conjugation chemistry, many biomolecules can now be routinely functionalized for effective and stable interaction with lipid biomembranes e.g. by conjugation of lipids to carbohydrates [164–166], peptides [73,137], peptide nucleic acids [167] and oligonucleotides (LiNAs) [40,168–170]. This strategy was shown to be effective with liposomes[73] and EVs [149]. Still, there are very few examples of multifunctional LNPs, and most are focused on surface modification with antibodies or co-encapsulation of siRNAs with hydrophobic chemotherapeutics which shows the importance of chemistry in the development of more commercially available building blocks for automated synthesis of peptides and oligonucleotides to accelerate application of reported chemistries in new combinations and formulations. Another important aspect is the potential for automatization and parallelization of complex LNPs during formulation development, since each functional component can be optimized separately and then combined to form the final formulation which may allows greatly accelerated drug discovery programmes by use of libraries which can be investigated in parallelized high-throughput combinatorial approaches including robotics and in-silico library design. Biodistribution and biophysical behaviour of LNPs is primarily dominated by the characteristics of surface lipids of the LNP, especially at low surface coverage, and to a lesser degree by the surface anchored functionalities and bioactive ON-conjugates which are small compared to e.g. 100 nm LNPs, except for the effect of specific surface attached receptor targeting ligands. The great potential of RNA therapeutics lies in the versatility of many different available targets and the possibility to address natural variability between patients, disease stages and cell types[171] and also heterogeneity in tissues by using multiple constructs for therapeutic approaches at the same time. With heterogeneity in cancer diseases as an example, we can see that single target approaches in drug development i.e. modulating levels of single genes or proteins, are often not effective enough and frequently elicit cellular compensatory mechanisms that result in drug resistance[172]. With great advances in genomics, transcriptomics, and proteomics, it is now possible to get a very detailed description of the heterogeneity at specific stages of the disease that allow individualized treatment recommendations and application of precise, combinatorial (multi-target) therapies [173]. In practice, there are not enough suitable therapeutics currently available, development of personalized drugs is still time consuming, often prohibitively expensive, and more generally, not sufficiently implemented in clinics and not routinely used with current regulatory standards for drug approval. Progress in our understanding of EVs and their therapeutic applications suggests that addressing complex diseases, like cancer, may require more complex multifunctional formulations which necessitate the development of multifunctional, complex formulations e.g. based on biomimetic LNPs, alongside more traditional ON-conjugates, especially in the transition period from currently available ON therapeutics to more personalized treatments. With inspiration from the field of engineering, creating complex formulations could be done in a more modular fashion, by developing interchangeable, cross-compatible subcomponents, such as libraries of lipidated entities, e.g., lipidated oligonucleotides, immunomodulatory peptides and specific protein targeting ligands, as well as non-toxic, adjustable lipidic carriers, like liposomes or biomimetic hybrid particles (e.g. liposome-EV hybrids). By exploiting strong partitioning of lipidated molecules to lipid bilayer membranes (as seen for natural processes of membrane anchoring on cell surfaces). The adjustment of the number of functionalities and individual concentration of active lipidated molecular entities results in defined relative ratios between different surface attached functionalities (which is difficult to achieve with classical conjugation strategies) to match disease specific demands and reduce overall toxicity. The suggested mix-and-match strategy may enable a highly parallelized and automated drug development process and present an enabling platform for the creation of truly personalized medicines, lowering costs and time from diagnosis to treatment and potentially improve prognosis for a large group of patients. The proposed strategies represent only a selection of possible solutions to some of the major challenges in oligonucleotide drug delivery and many may face multiple setbacks before becoming a clinical reality. Especially nanoparticle-based formulations need to deal with batch-to-batch variability, development of standardized formulation and manufacturing protocols that are scalable and economical, address logistical points of stability, storage, and handling between manufacturing and clinical application. Increasing number of components in complex formulations complicate many key steps in upscaling and production and add additional challenges during clinical trials, where multifunctional drugs will need to be assessed for broad range of potential side effects for both the bioactive oligonucleotides and auxiliary formulation components. In summary, with an increasing number of approved ON- therapeutics and therapies based on biologicals, together with advancements in nanoparticle-based formulations and bioconjugation, more oligonucleotide therapeutics are likely to be improved and the continuous development of safe and more complex formulations will enable the treatment of diseases where efficient therapies are still limited by insufficient delivery to the tissue or organ of interest.

## Figures and Tables

**Table:** 

ON	oligonucleotide
PNA	peptide nucleic acid
PMO	phosphorodiamidate morpholino oligomers
RNAi	RNA interference
2’-OMe	2’-O-methyl
2’-MOE	2’-O-methoxyethyl
LNA	locked nucleic acid
LNP	lipid nanoparticles
ASO	antisense oligonucleotides
siRNA	small interfering RNA
BBB	blood-brain-barrier
ASGPR	asialoglycoprotein receptor
GalNAc	N-acetyl galactosamine
PEG	polyethylen glycol
CuAAC	Cu-assisted azide-alkyne cycloaddition
FR	folate receptor
Dox	doxorubicin
M6P	mannose-6-phosphate
LiNA	Lipidated oligonucleotides
Chol	cholesterol
C12	lauric acid
C16	palmitic acid
DsiRNA	Dicer-substrate siRNA
PE	phosphothioethanol
POPC	palmitoyl-oleyl-phosphatidylcholine
CPP	cell-penetrating peptide
EPA	eicosapentaenoic acid
DHA	docosahexaenoic acid
LA	lithocholic acid
PC	phosphocholine
DCA	docosanoic acid
EV	extracellular vesicle
HA	hemagglutinin envelope protein
Ahx or X	6-aminohexanoic acid
B	β-alanine
RA	rheumatoid arthritis
Ab	antibody
ALL	acute lymphoblastic leukemia
PSMA	prostate-specific membrane antigen
HER	human epidermal growth factor receptor
DOTMA	1,2-di-O-octadecenyl-3- trimethylammonium-propane
DOTAP	1,2-dioleoyl-3-trimethylammonium-propane
DOPE	1,2-dioleoyl-sn-3-phosphoethanolamine
DOPC	dioleoyl phosphatidylcholine
DODMA	1,2-dioleyloxy-*N,N*-dimethyl-3-aminopropane
DSPC	1,2-distearoyl-sn-glycero-3-phosphocholine
DLin-MC3-DMA	heptatriaconta-6,9,28,31-tetraen-19-yl-4-(dimethylamino) butanoate
SM-102	heptadecan-9-yl-8-((2-hydroxyethyl)(6-oxo-6-(undecyloxy)hexyl)amino)octanoate
*Htt*	Huntingtin gene
